# Supplementary material for: Evolution of a flipped pathway creates metabolic innovation in tomato trichomes through BAHD enzyme promiscuity
Source: Nat Commun. 2017 Dec 12;8:2080. doi: 10.1038/s41467-017-02045-7 (PMC5727100; doi:10.1038/s41467-017-02045-7)
Supplement: Supplementary file 1 — Supplementary Information [file 41467_2017_2045_MOESM1_ESM.pdf]

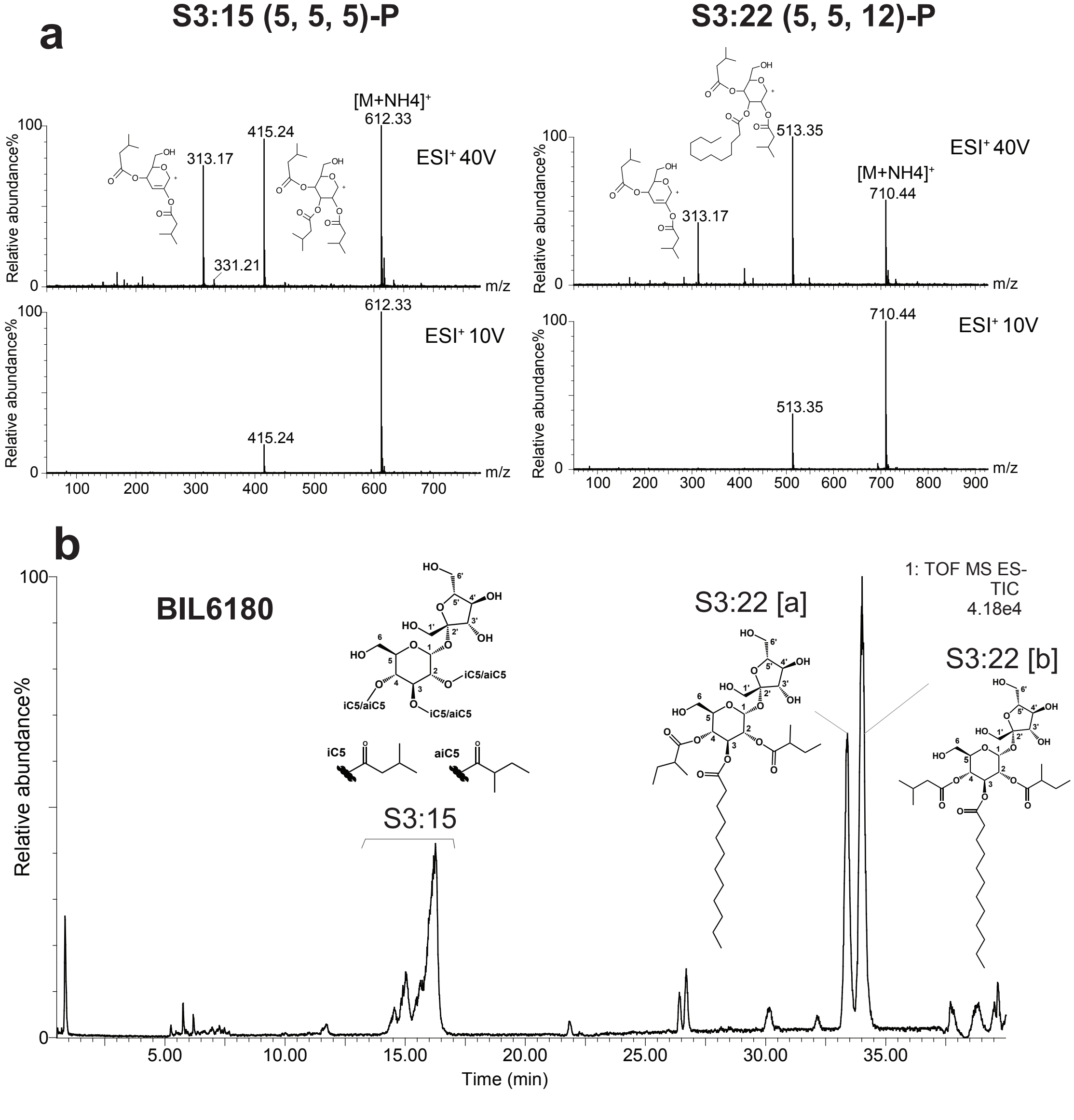

Supplementary Figure 1. BIL6180 plants produce major triacylsucroses with all acyl chains on one ring. (**a**) The BIL6180 acylsucroses S3:15 (5,5,5) and S3:22 (5,5,12) showed the positive mode MS fragments of *m/z* 415.2 and *m/z* 513.3, respectively. These correspond to three C5 acyl chains on one ring for *m/z* 415.2 and C5, C5, C12 on one ring for *m/z* 513.3. (**b**) 40 min LC reverse phase separation revealed that the major BIL6180 S3:15 and S3:22 acylsucrose peaks are composed of isomers that have iC5 or aiC5 chains at position R_2 (NMR characterization data for S3:15, S3:22[a], and S3:22[b] are shown in Methods).

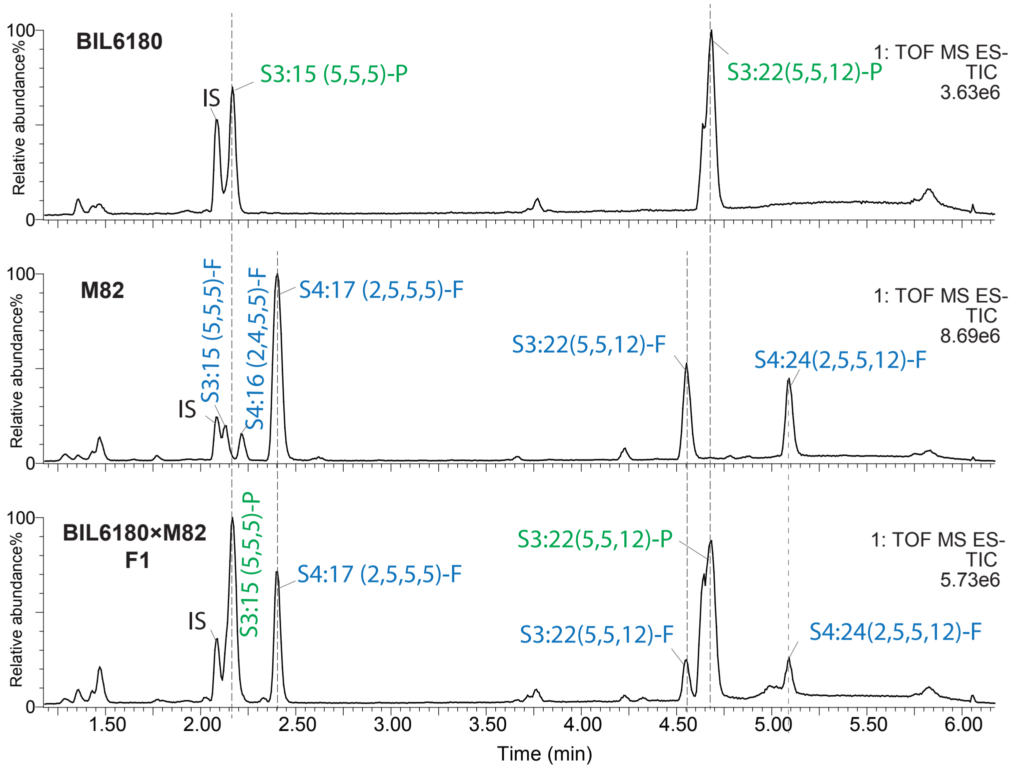

Supplementary Figure 2. Accumulation of both F- and P-type acylsucroses in BIL6180 × M82 F1 plants. The F1 plants contain a mixture of peaks that have the same chromatographic retention time with the major P-type acylsucroses S3:15-P and S3:22-P (green) found in BIL6180 and the F-type acylsucroses S4:17-F, S3:22-F, and S4:24-F (blue) that accumulate in M82. IS = internal standard.

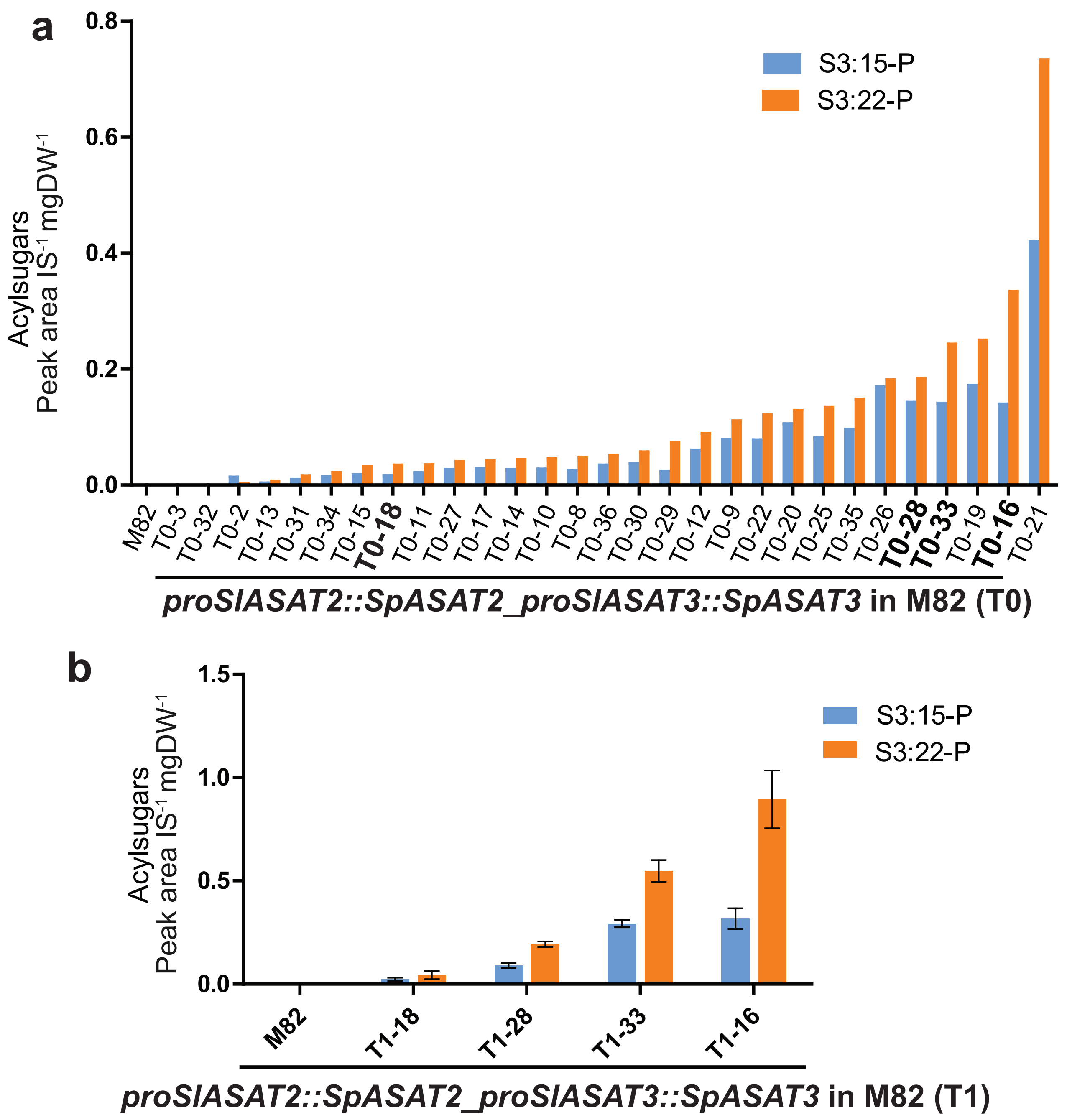

Supplementary Figure 3. Transgenic plants expressing both *Sp-ASAT2* and *Sp-ASAT3* in trichomes of cultivated tomato *S. lycopersicum* M82 produce the P-type acylsucroses S3:15-P and S3:22-P. (**a**) In each independent transgenic T0 line, the extracted ion chromatogram peak areas of S3:15-P and S3:22-P were divided by internal standard (IS) peak areas and normalized by leaf dry weight (DW). Each pair of bars represents results from an independent transgenic event. Both S3:15-P and S3:22-P were not detected in extracts of the parent M82 plant. T0 lines that are used for further analysis in the T1 generation are in bold font. (**b**) In T1 generation, extracted ion chromatogram peak areas of S3:15-P and S3:22-P divided by IS peak areas normalized by leaf DW are shown for four independent lines. Five transgenic plants genotyped by PCR from each independent T1 transgenic line are used for analysis with s.e.m. shown.

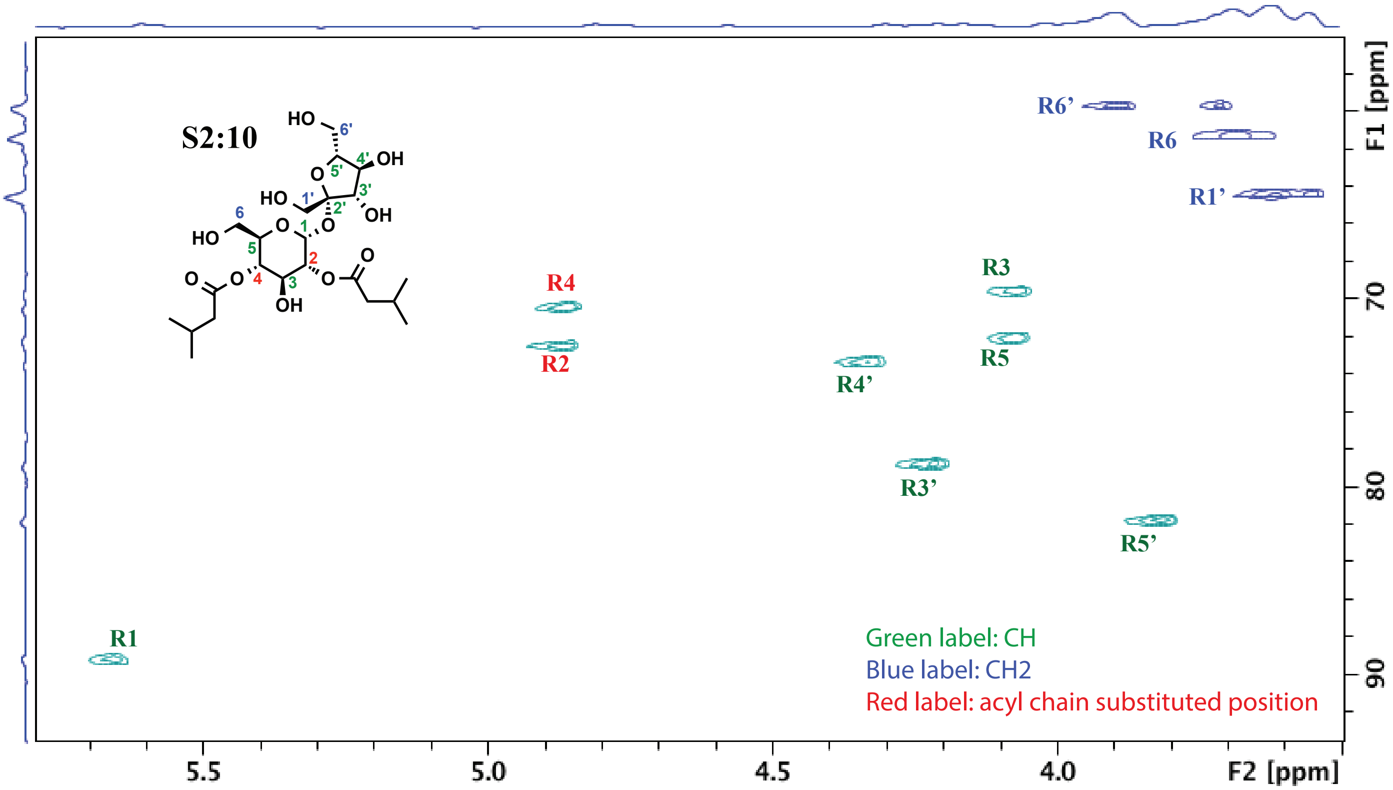

Supplementary Figure 4. The HSQC NMR spectrum of the diacylsucrose S2:10 (iC5, iC5) showing resonances of the sucrose core. The HSQC spectrum has downfield proton chemical shifts that indicate acylation at the sucrose R_2 and R_4 positions.

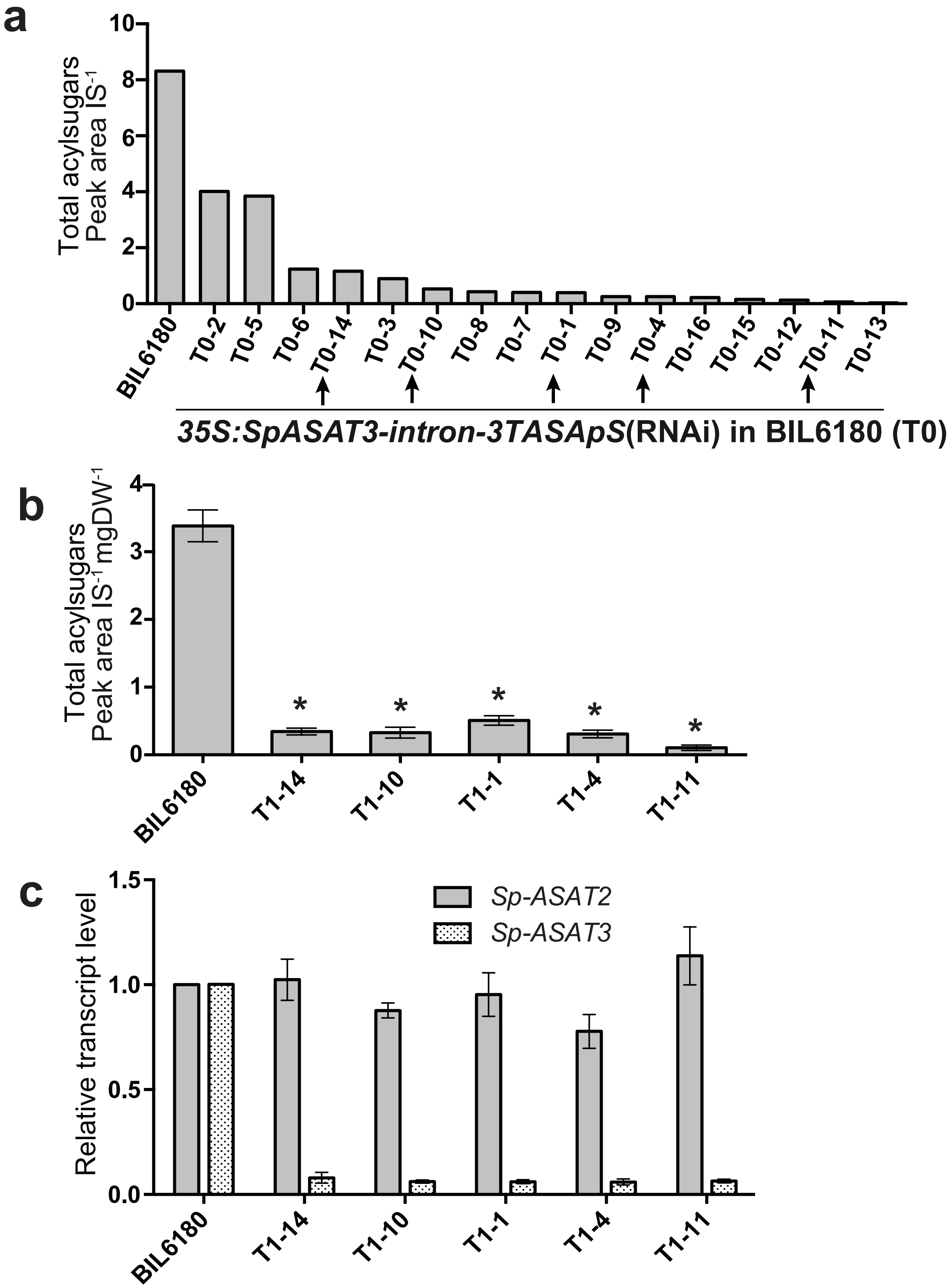

Supplementary Figure 5. Suppression of *Sp-ASAT3* in BIL6180 by RNAi led to reduction of tomato total acylsugar peak areas and down regulation of *Sp-ASAT3* expressions in the transgenic plants. (**a**) Summed extracted ion chromatogram peak areas divided by internal standard (IS) peak areas for all detectable acylsugars are shown for each T0 independent transgenic plant. T0 lines that are used for further analysis in the T1 generation are indicated by arrows. (**b**) Summed extracted ion chromatogram peak areas divided by IS peak areas for all detectable acylsugars normalized by leaf dry weight (DW) are shown for five independent T1 transgenic lines. **P* < 0.01; relative to BIL6180; two-tailed unpaired *t*-test. (**c**) Relative transcript levels for *Sp-ASAT2* and *Sp-ASAT3* determined by Real-time PCR are shown for the corresponding T1 transgenic plants in **b**. Five transgenic plants genotyped by PCR from each T1 transgenic line are used for analysis with s.e.m. shown.

a

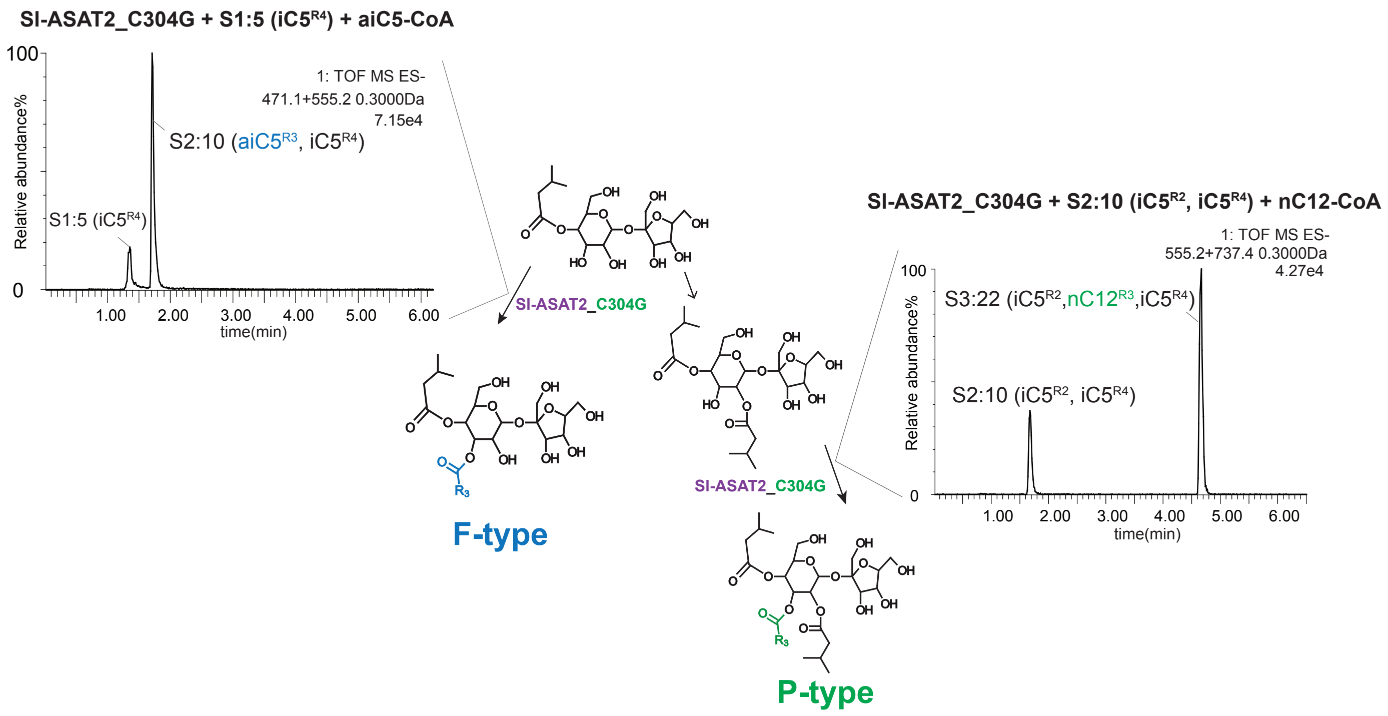

b

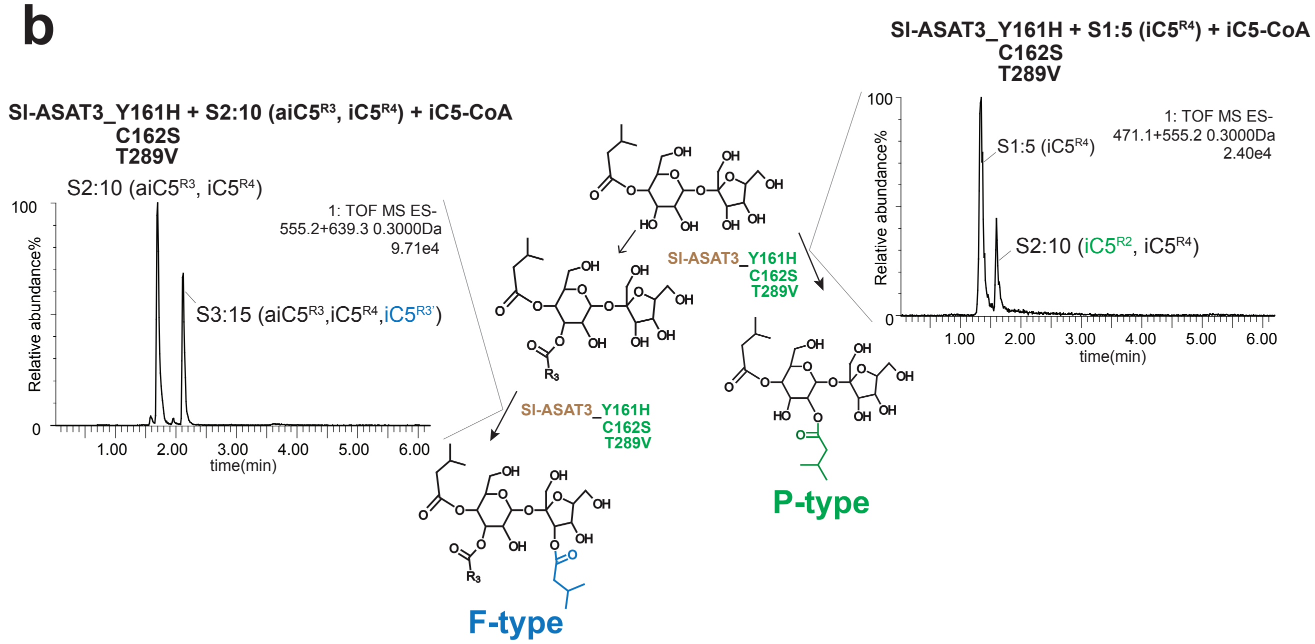

Supplementary Figure 6. Amino acids that contribute to Sl-ASAT2 and Sl-ASAT3 promiscuity revealed by site directed mutagenesis. (**a**) A mix of F- and P-type activity results from the C304G substitution in the F-type Sl-ASAT2. (**b**) Both F- and P-type activity results from three residue substitutions (Y161H, C162S, and T289V) in the F-type Sl-ASAT3 enzyme.

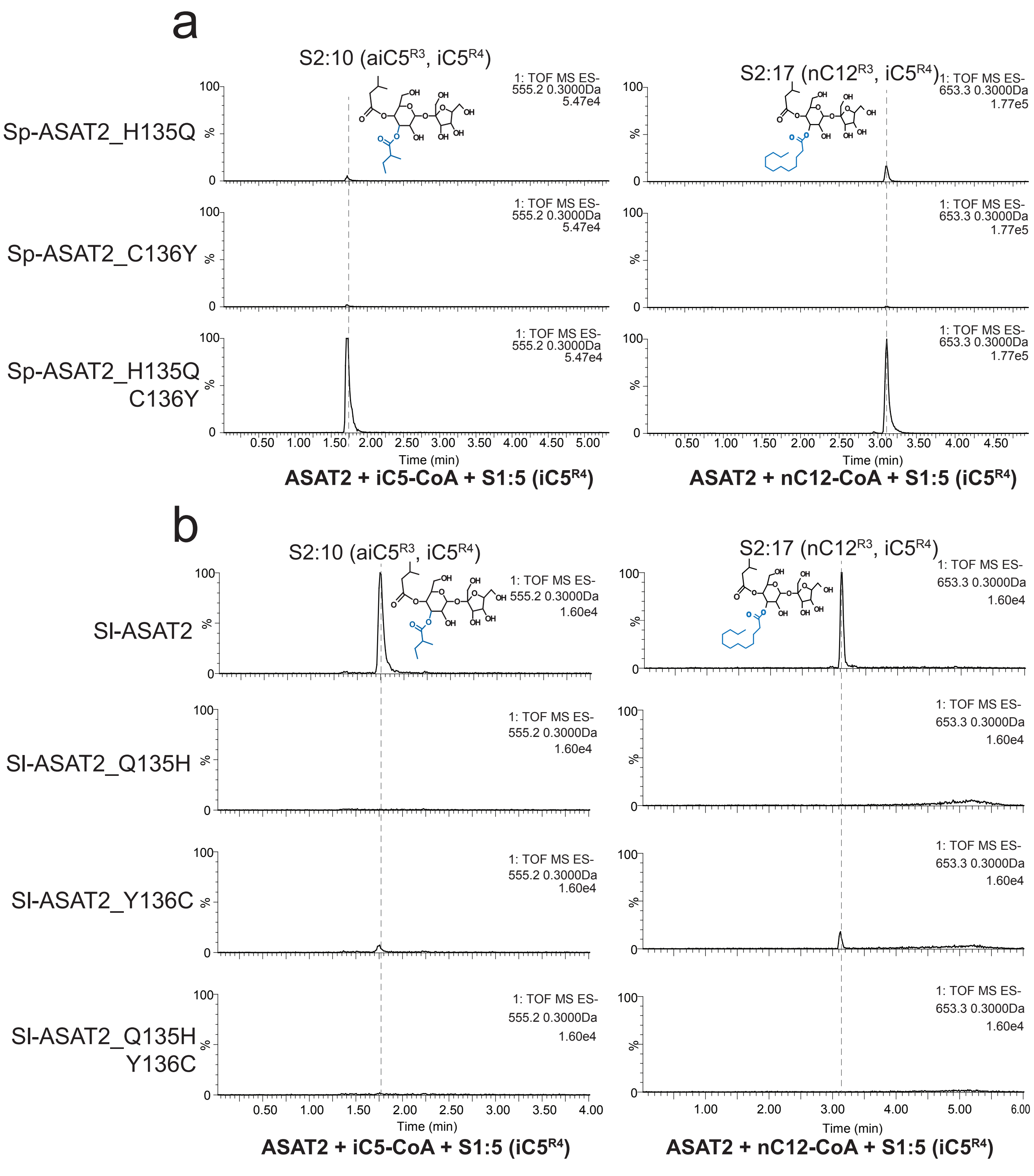

Supplementary Figure 7. Q[135] and Y[136] contribute to conversion of P-type Sp-ASAT2 to F-type *in vitro* activity. (**a**) Sp-ASAT2 with either single residue substitution H135Q or C136Y produce minor amounts of diacylsucrose *in vitro* products, while the H135Q and C136Y double mutant protein gains strong F-type activity. (**b**) The Sl-ASAT2 with amino acid substitution Q135H or Y136C, or double mutant Q135H and Y136C reduced the F-type *in vitro* diacylsucrose product peak compared with Sl-ASAT2. The Y-axes are relative abundance of the chromatogram peak intensity.

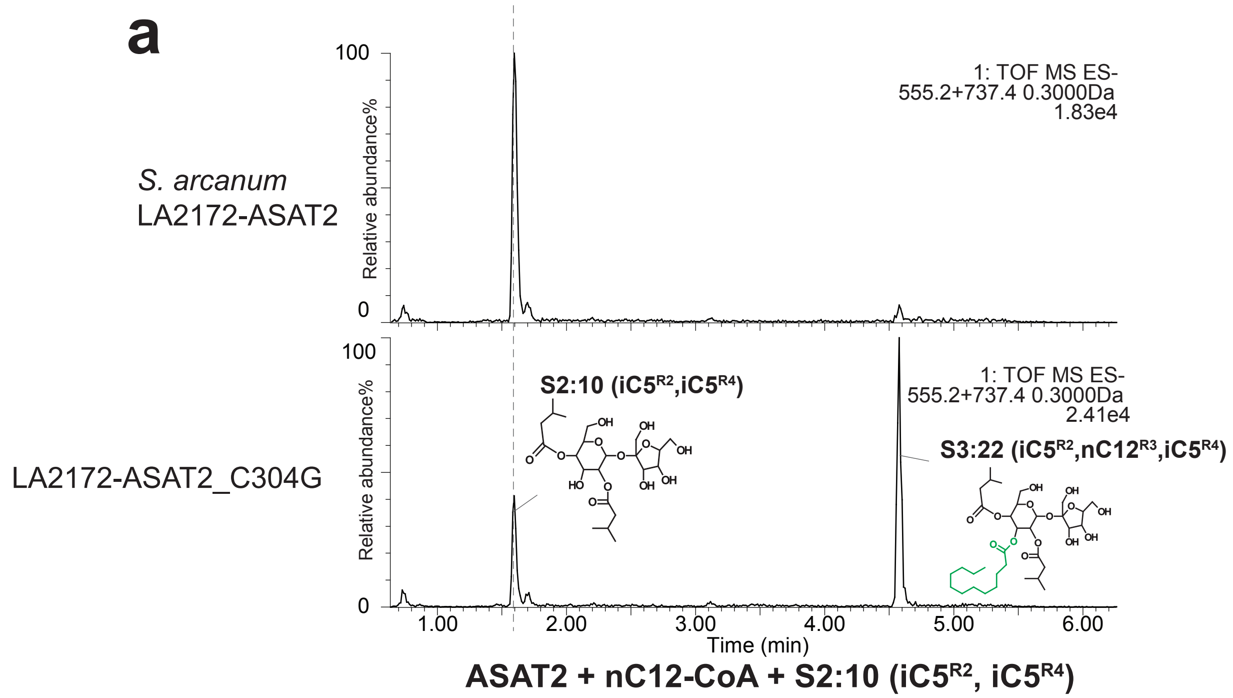

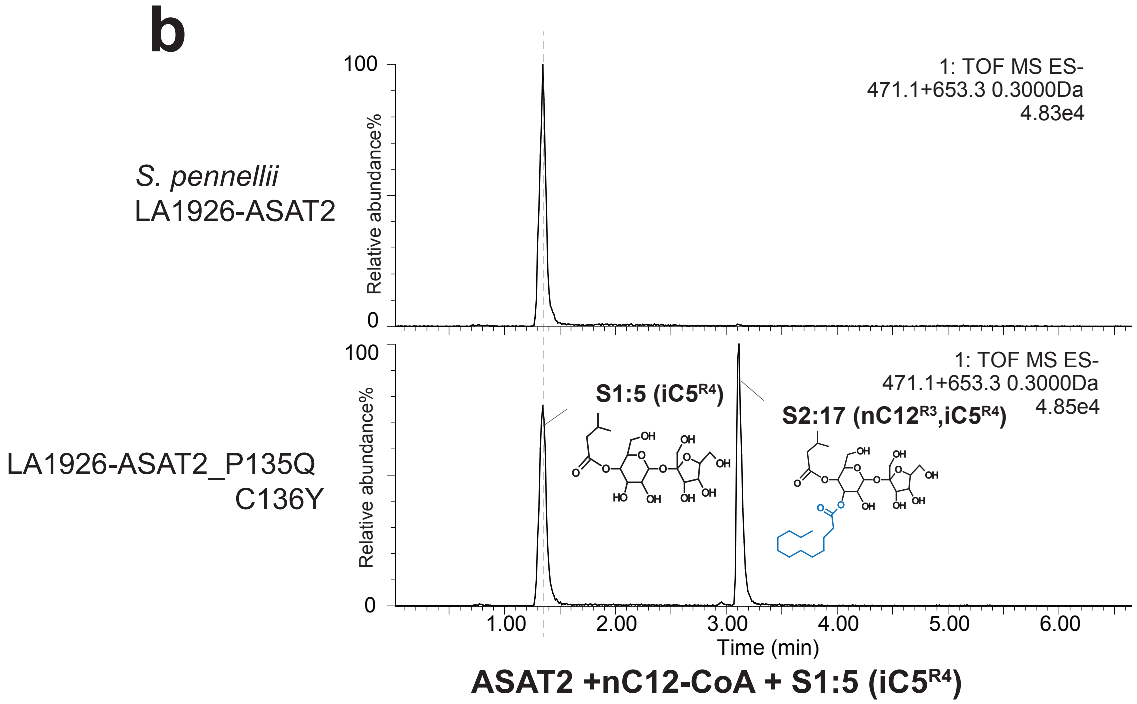

Supplementary Figure 8. Test of key residues that influence F- or P-type ASAT2 activities in two additional wild tomato isoforms. (**a**) The F-type *S. arcanum* LA2172-ASAT2 with the C304G substitution acquired the P-type activity to produce the S3:22 ($iC5^{R2}$, $nC12^{R3}$, $iC5^{R4}$) triacylsucrose. (**b**) The P-type *S. pennelii* LA1926-ASAT2 with P135Q and C136Y double substitutions acquired the F-type activity and produced the S2:10 ($aiC5^{R3}$, $iC5^{R4}$) diacylsucrose.

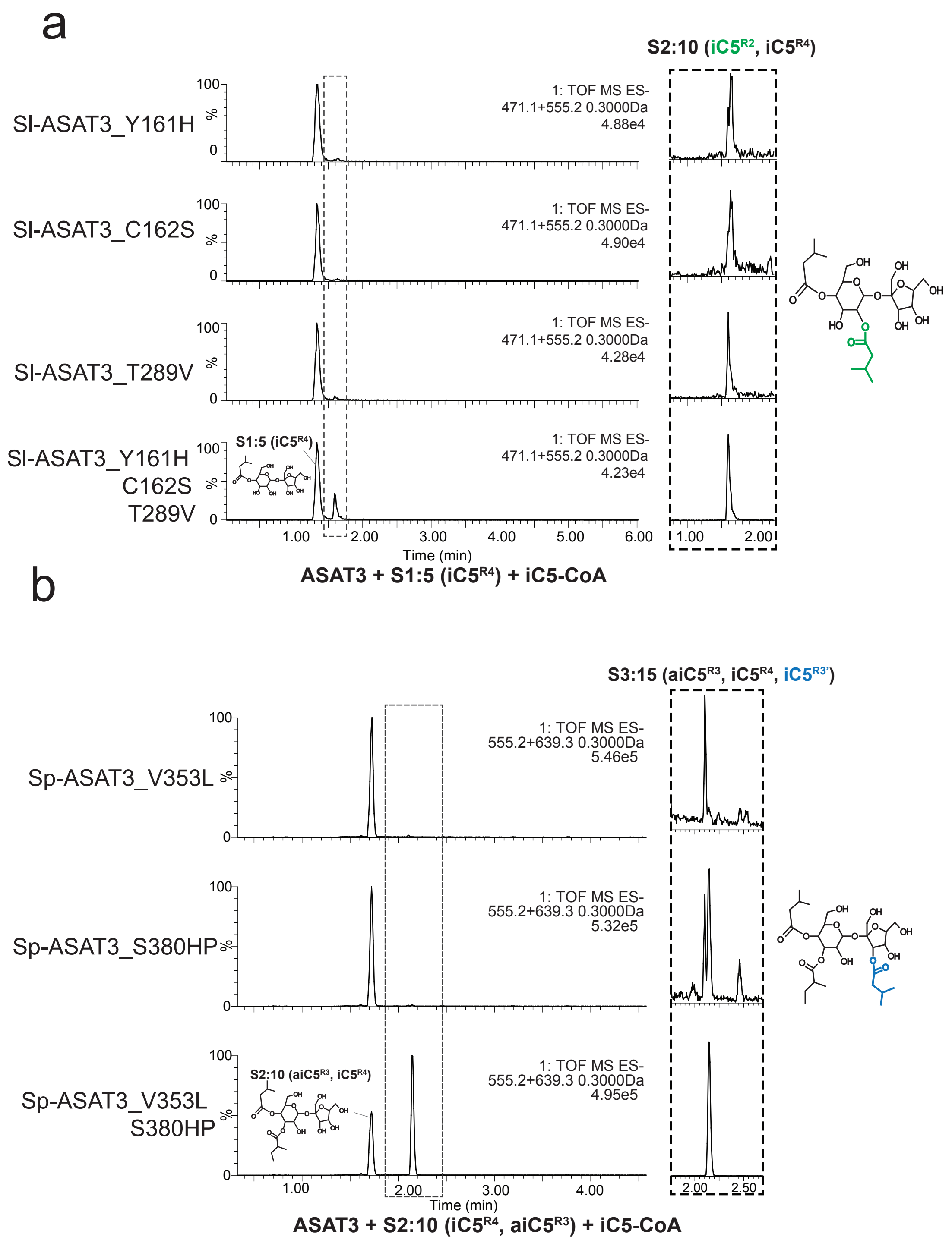

Supplementary Figure 9. Test of amino acids for their influence on ASAT3 P- or F-type *in vitro* activities. (**a**) Each Sl-ASAT3 single mutant (Y161H, C162S, T289V) produced a minor amount of diacylsucrose product S2:10 ($iC5^{R2}$, $iC5^{R4}$) *in vitro*, while combining the changes into a triple Sl-ASAT3 mutant led to increased activity. (**b**) The Sp-ASAT3 mutants with residue substitutions V353L or S380HP produced minor amounts of the triacylsucrose product S3:15 ($aiC5^{R3}$, $iC5^{R4}$, $iC5^{R3'}$) *in vitro*, while the combination of mutations caused dramatically increased F-type activity. The images in the boxes on the right of each chromatogram are close-up images of the boxed in regions to the left. The Y-axes are relative abundance of the chromatogram peak intensity.

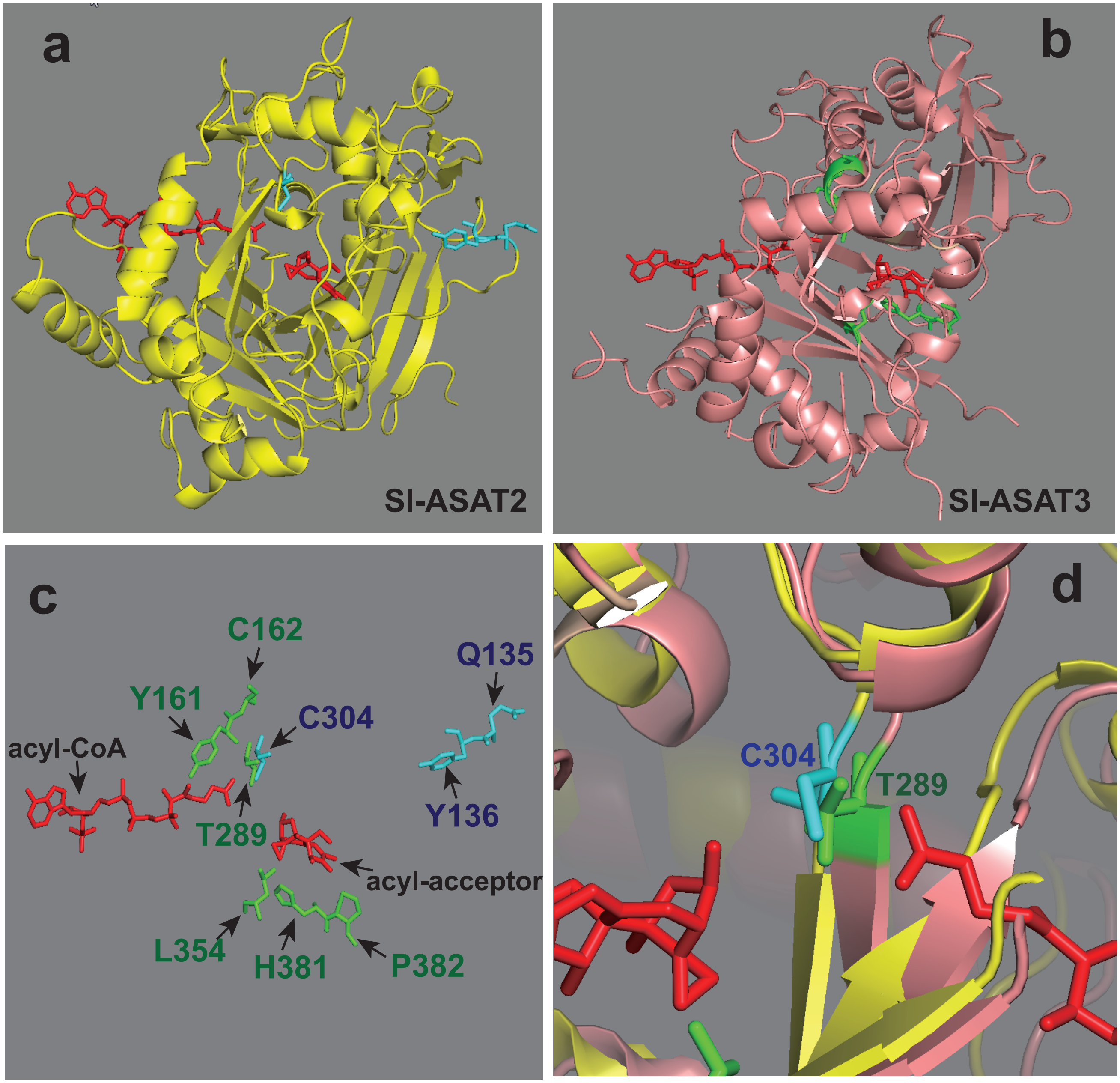

Supplementary Figure 10. Homology modeling places residues affecting Sl-ASAT2 and Sl-ASAT3 activities surrounding the substrate binding sites. (**a**) and (**b**), The predicted structures of Sl-ASAT2 in yellow (a) or Sl-ASAT3 in pink (b) were modeled based on the trichothecene 3-*O*-acetyltransferase —acyl-CoA 3B2S structure[1]. The residues identified as being involved in specifying P- versus F- activities are featured in blue (Sl-ASAT2) and green (Sl-ASAT3). The acyl-CoA donor and acyl acceptor from the 3B2S structure are shown in red. (**c**) Close up of the modeled positions of the residues featured in **a** and **b** are superimposed on the 3B2S structure. (**d**) A close up of the residues Cys^{304} in Sl-ASAT2 and Thr^{289} in Sl-ASAT3, showing that they are predicted to occupy similar positions in the respective 3D structures.

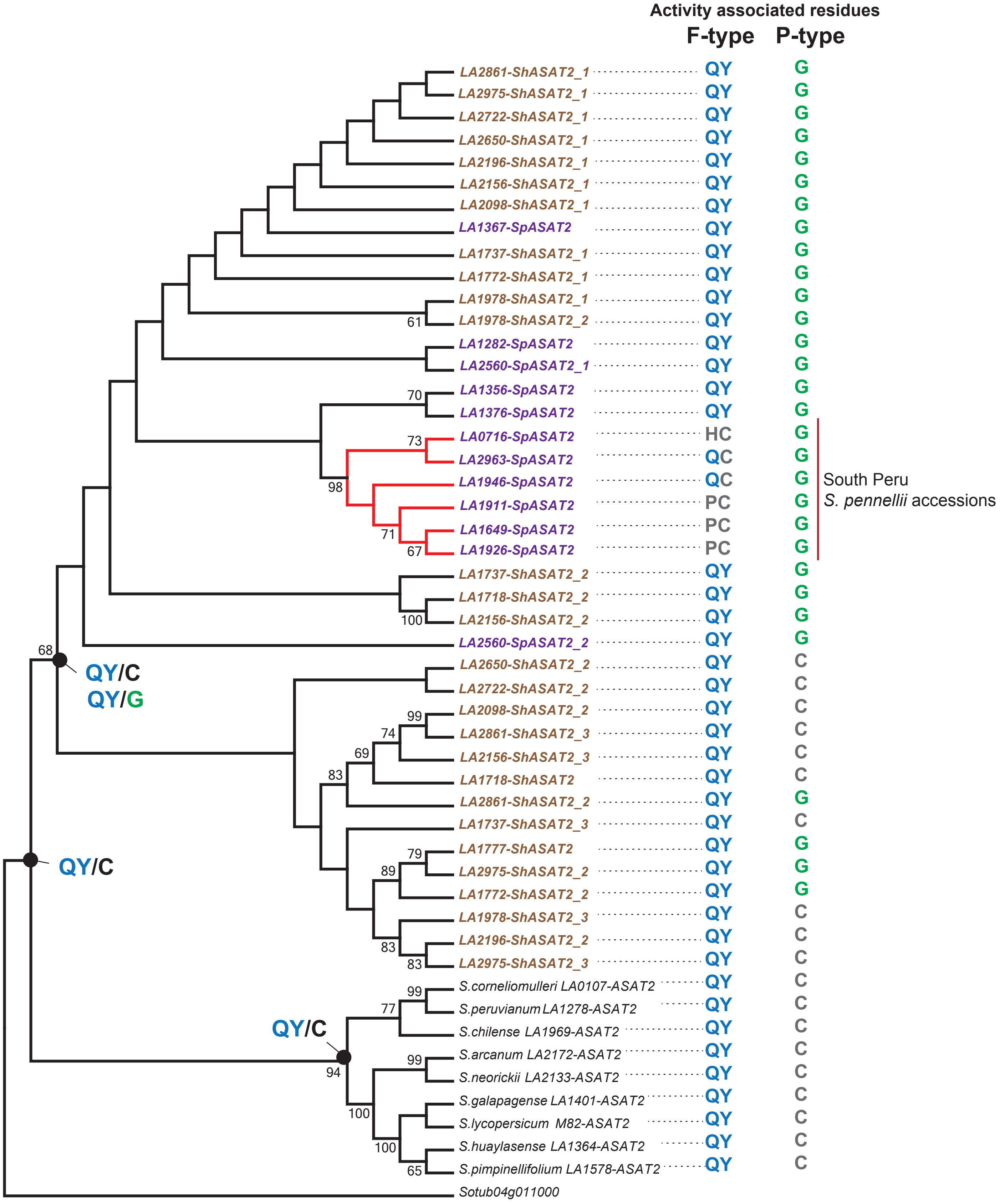

Supplementary Figure 11. Maximum likelihood phylogenetic tree obtained using the nucleotide sequences of *ASAT2* variants cloned from different tomato species and accessions. The *ASAT2* variants from *S. pennellii* and *S. habrochaites* are in purple and brown, respectively. The amino acids at the positions that influence enzyme types are listed: F- and P-type residues are colored with blue and green. The *S. pennellii ASAT2* lineage in red is from accessions collected in southern Peru; these are notable because they lack the QY residues associated with F-type activity found in all other tomato Solanum enzymes. The ancestral states of the activity-associated residues are based upon extant species sequences. The potato *ASAT2* homologue *Sotub04g011000* serves as outgroup. The bootstrap values were obtained after 1000 replicates. The nodes with bootstrap values lower than 60 were not shown.

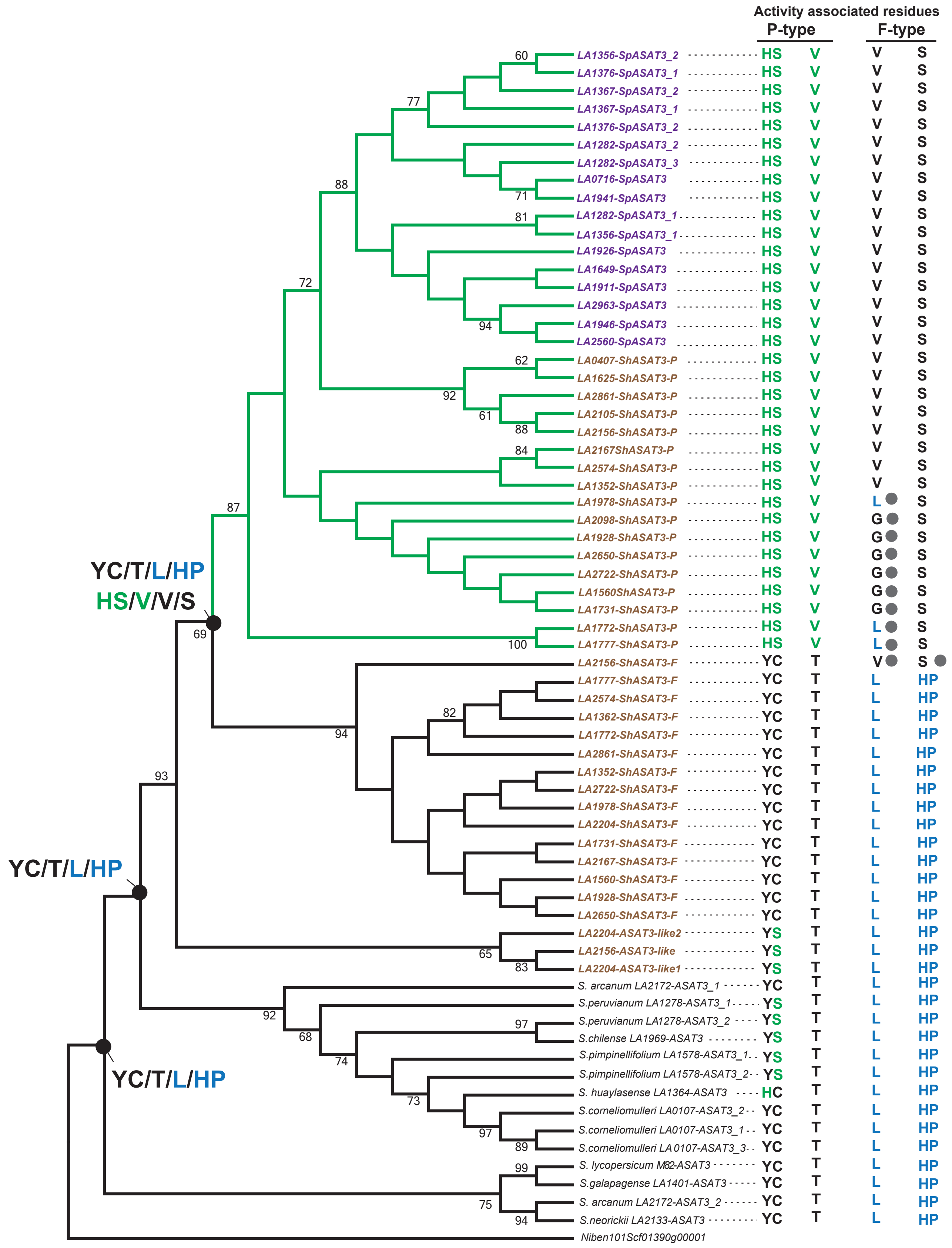

Supplementary Figure 12. Maximum likelihood phylogenetic tree obtained using the nucleotide sequences of *ASAT3* variants cloned from different tomato species and accessions. The *ASAT3* variants from *S. pennellii* and *S. habrochaites* are in purple and brown, respectively. The amino acids at the positions associated with F- or P-type activities are listed. The F- and P-type residues are in blue and green, respectively. *S. habrochaites ASAT3* sequences are from a published study[2]. Gray circles indicate residues in Sh-ASAT3-P not associating with F-type activity. The tobacco *ASAT3* homologue *Niben101Scf01390g00001* serves as outgroup. The bootstrap values were obtained after 1000 replicates. The nodes with bootstrap values lower than 60 were not shown.

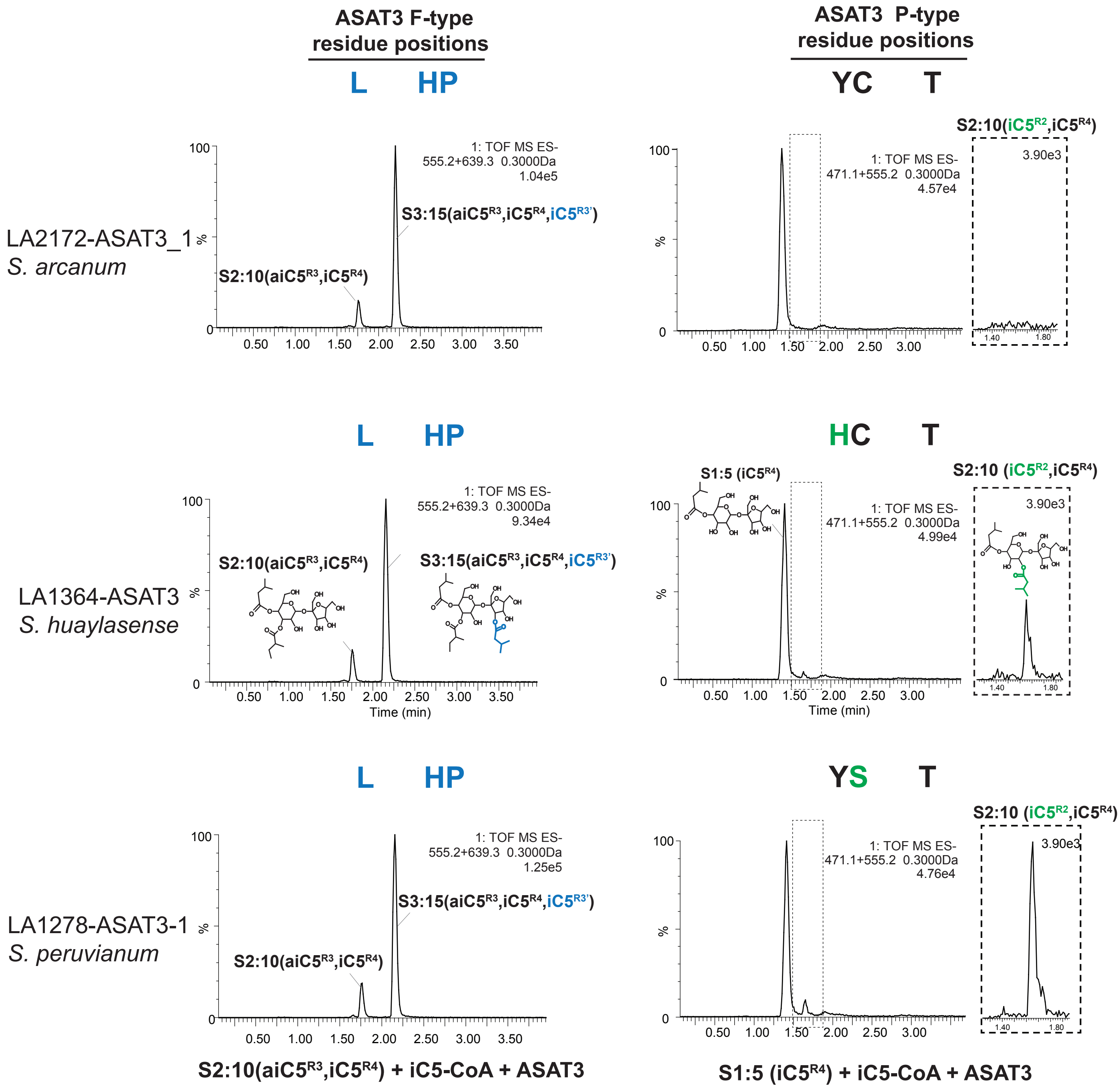

Supplementary Figure 13. Test of natural variant ASAT3 F- and P-type *in vitro* enzyme activities from the wild tomato species *S. arcanum* (LA2172-ASAT3_1), *S. huaylasense* (LA1364-ASAT3), and *S. peruvianum* (LA1278-ASAT3_1). Chromatograms of assays using the ASAT3 F-type acyl acceptor substrate S2:10 (aiC5^{R3}, iC5^{R4}) and measuring the product S3:15 (aiC5^{R3}, iC5^{R4}, iC5$^{R3'}$) are in the left column. Chromatograms of assays using the ASAT3 P-type acyl acceptor substrate S1:5 (iC5^{R4}) and measuring product S2:10 (iC5^{R2}, iC5^{R4}) are shown on the right. The boxed regions are shown to the right of the P-type assays magnified approximately 10-fold. The amino acids associated with ASAT3 F- and P-type activities are highlighted with blue and green colors, respectively. The Y-axes are relative abundance of the chromatogram peak intensity.

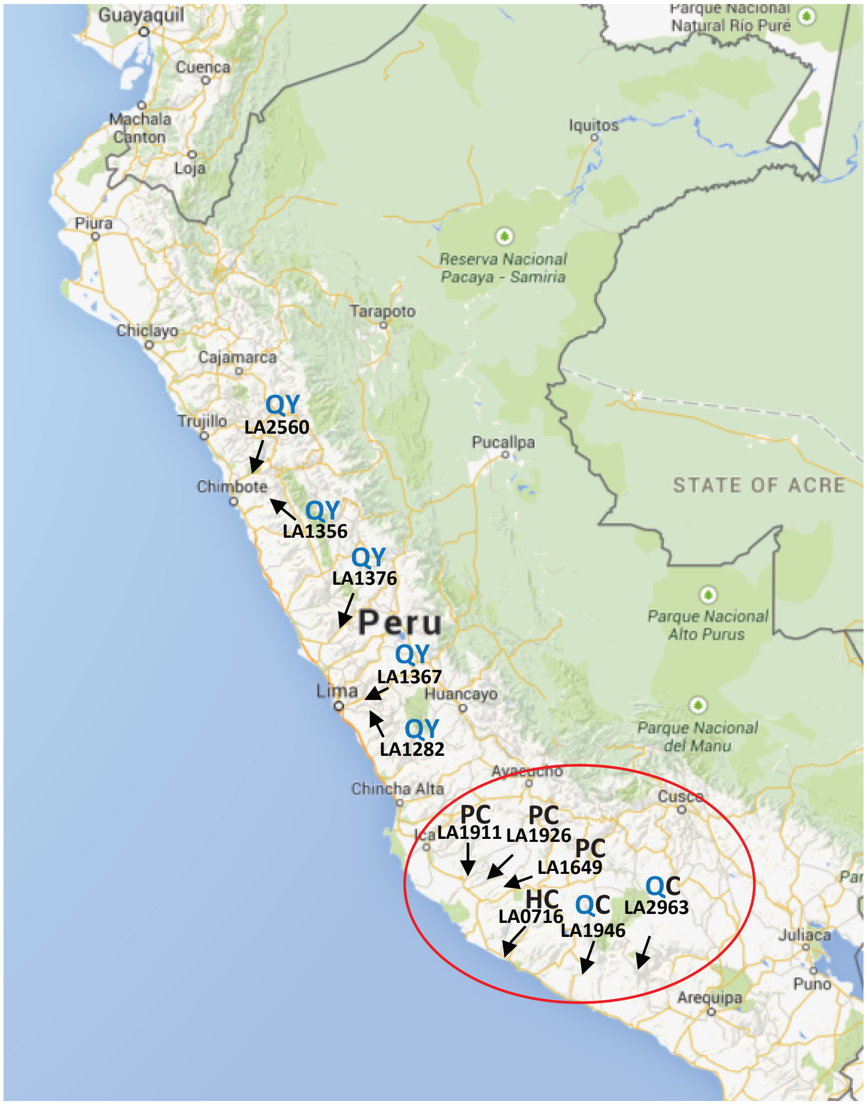

Supplementary Figure 14. Geographic locations of *S. pennellii* ASAT2 types. Accessions containing ASAT2 with F and P activities (with amino acids $Q^{135}Y^{136}$) are from Lima and north. In contrast, ASAT2-P lacking F-type activity (with amino acids $H^{135}C^{136}$, $P^{135}C^{136}$ and $Q^{135}C^{136}$) are from the southern area of the range; a red ellipse highlights these accessions.

Supplementary Table 1. Primers used in this study.

Name	Primers (5' to 3')	Purpose
pENTR-att-gg4F	CACGCAAGGTCTCCAACCCAGCTTTCTTGTACAA	Amplify pENTR/D-TOPO fragment for Golden Gate Assembly
pENTR-att-gg4R	CACGCAAGGTCTCCGAGCCTGCTTTTTTGTACAAA	
SlASAT2-pro-gg4F	CACGCAAGGTCTCCGCTCCTCAATTATCCATTCGTCTC	Amplify *Sl-ASAT2* promoter region for Golden Gate Assembly
SlASAT2-pro-gg4R	CACGCAAGGTCTCCCATATTTTGTTTGATACAAATTTTGTTTGA	
SpASAT2with1kb-gg4F	CACGCAAGGTCTCGTATGAGTAGTGTATCAAGACTTGT	Amplify *Sp-ASAT2* and 1kb downstream of *Sp-ASAT2* for Golden Gate Assembly
SpASAT2with1kb-gg4R	CACGCAAGGTCTCGTTAGCTTTGTACATCCTTCAAATAATTAAGA	
SlASAT3-pro-gg4F	CACGCAAGGTCTCCCTAACTTGCAAATATAACTTTAGTTATTTAT	Amplify *Sl-ASAT3* promoter region for Golden Gate Assembly
SlASAT3-pro-gg4R	CACGCAAGGTCTCCgccatTTTAGCTATCAAAAAAATTAATATAAATTAAGC	
SpASAT3-gg4F	CACGCAAGGTCTCCtggcaTCATCAACAATTATATCTAG	Amplify *Sp-ASAT3* for Golden Gate Assembly
SpASAT3-gg4R	CACGCAAGGTCTCCGGTttatttGGTTGATTCAACAACTG	
Sp11AT3-RNAi-F	CACCCATAAACGATTGGGCGTCTACAGC	Amplify the fragment of *Sp-ASAT3* to be recombined into pHELLSGATE12 for RNAi silencing
Sp11AT3-RNAi-R	TTTAGCCCATCTCCACTTTGTTC	
ASAT2-C304G_F	GGGAAACATTggtTCTCTATTTTC	Site-directed mutagenesis of C304G in ASAT2
ASAT2-C304G_R	ATAGTGTGTTTGGGCAATG	
ASAT2-G304C_F	GGGAAACATTtgtTCTCTATTTTCTATAC	Site-directed mutagenesis of G304C in ASAT2
ASAT2-G304C_R	ATAGTGTGTTTGGGCAATG	
SpASAT2_HC135QY_F	TTAATAATCAgtaTACATACGAGGGTAG	Site-directed mutagenesis of both H135Q and C136Y in ASAT2
SpASAT2_HC135QY_R	TAACTTCTGTCGGATACAC	
SpASAT2_H134Q_F	TTAATAATCAgTGTACATACGAGG	Site-directed mutagenesis of H135Q in ASAT2
SpASAT2_H134Q_R	TAACTTCTGTCGGATACAC	
SpASAT2_C135Y_F	AATAATCACTaTACATACGAGGG	Site-directed mutagenesis of C136Y in ASAT2
SpASAT2_C135Y_R	AATAACTTCTGTCGGATACAC	

Name	Primers (5' to 3')	Purpose
SlASAT3-T289V_F	GGGAAATGCAgtATGTATCATTCTCAC	Site-directed mutagenesis of T289V in ASAT3
SlASAT3-T289V_R	ATTGTGTTCAGTGGAATTG	
SlASAT3_Y161H_F	TTTTGATGGAcATTGTCTCTCTAAATTC	Site-directed mutagenesis of Y161H in ASAT3
SlASAT3_Y161H_R	ATTGTATGTGATGTACATGC	
SlASAT3_C162S_F	GATGGATATTcTCTCTCTAAATTCATAAAC	Site-directed mutagenesis of C162S in ASAT3
SlASAT3_C162S_R	AAAAATTGTATGTGATGTACATG	
SlASAT3-YC161HS_F	TTTTGATGGAcattctCTCTCTAAATTCATAAAC	Site-directed mutagenesis of both Y161H and C162S in ASAT3
SlASAT3-YC161HS_R	ATTGTATGTGATGTACATGC	
SpASAT3-V288T_F	GGGAAATGCAacATGTATCATTCTCAC	Site-directed mutagenesis of V288T in ASAT3
SpASAT3-V288T_R	ATTGTGTTTAGTGGAATTGG	
SpASAT3-HS161YC_F	TTTTGATGGAtattgtCTCTCTAAATTCATAAATG	Site-directed mutagenesis of both H161Y and S162C in ASAT3
SpASAT3-HS161YC_R	ATTGTATGTGATGTACATGC	
SpASAT3_S380HP_F	CCCTAGCAACacatcCAAATAAGAACAACTTC	Site-directed mutagenesis of S380HP in ASAT3
SpASAT3_S380HP_R	TTACTCTTACAGGTTCACC	
SpASAT3_V353L_F	TGATGTTTATcTTTGCTCAGGCATG	Site-directed mutagenesis of V353L in ASAT3
SpASAT3_V353L_R	TGTGTATCCTTCTCTATTATGTTC	
SpASAT2-NheI-F	CTGGCTAGCATGAGTAGTGTATCAAGACTTGTAT	Amplify *ASAT2* from different *S. pennellii* accessions
SpASAT2-XhoI-R	CCGCTCGAGTTAGCTAGTTGGGGAGGCAAATTGT	
ShASAT2-NheI-F	CTGGCTAGCATGAGTAGTGTATCAAGACTTGTAT	Amplify *ASAT2* from different *S. habrochaites* accessions
ShASAT2-XhoI-R	CCGCTCGAGTTAGCTAGTTGGAGAGGCAAATC	
ASAT3-P_NheI-F	ctaGCTAGCATGGCATCATCAACAATTAT	P- primer to amplify *ASAT3* from different wild tomatoes
ASAT3-F_NheI-F	ctaGCTAGCATGGCATCATCAAAAATGATATCT	F- primer to amplify *ASAT3* from different wild tomatoes
ASAT3-Xho1-R	ccgCTCGAGTTATTTGGTTGATTCAACAACTGG	Reverse primer to amplify *ASAT3* from different wild tomatoes

Name	Primers (5' to 3')	Purpose
pK7WG_F	TTGACTCTAGCTAGAGTCCGAA	Primers used to genotype the transgenic plants carrying the vector pK7WG.
pK7WG-R	ATTGAACAAGATGGATTGCACGCA	
pHELLSGATE_F	AGAAAGTATCCATCATGGCTGATG	Primers used to genotype the transgenic plants carrying the vector pHELLSGATE12
pHELLSGATE_R	TCCAACGAGCATAATTCTTCAGA	
SpASAT2_qRT-F	ACATACGAGGGTAGTCTTGCAG	Real-time PCR primers for *Sp-ASAT2*
SpASAT2_qRT-R	GTTCATGAAGTTGCCGAACGT	
SpASAT3_qRT-F	GCTGTGGGTGTAGTCTTCCC	Real-time PCR primers for *Sp-ASAT3*
SpASAT3_qRT-R	CCATGTTTCGAGCTGTAGACG	
EF-1a_qRT-F	AGCTTCACTGCCCAGGTCATCATC	Real-time PCR primers for the elongation factor gene *Solyc06g005060*
EF-1a_qRT-R	TGGGCTTGGTGGGAATCATCTTA	

Supplementary References

1. Garvey, G. S., McCormick, S. P. & Rayment, I. Structural and functional characterization of the TRI101 trichothecene 3-*O*-acetyltransferase from *Fusarium sporotrichioides* and *Fusarium graminearum*: Kinetic insights to combating Fusarium head blight. *J. Biol. Chem.* **283,** 1660–1669 (2008).
2. Schilmiller, A. L. *et al.* Functionally divergent alleles and duplicated loci encoding an acyltransferase contribute to acylsugar metabolite diversity in *Solanum* trichomes. *Plant Cell* **27,** 1002–1017 (2015).
